# *S*[+] Apomorphine is a CNS penetrating activator of the Nrf2-ARE pathway with activity in mouse and patient fibroblast models of amyotrophic lateral sclerosis^☆^

**DOI:** 10.1016/j.freeradbiomed.2013.04.018

**Published:** 2013-08

**Authors:** Richard J. Mead, Adrian Higginbottom, Scott P. Allen, Janine Kirby, Ellen Bennett, Siân C. Barber, Paul R. Heath, Antonio Coluccia, Neelam Patel, Iain Gardner, Andrea Brancale, Andrew J. Grierson, Pamela J. Shaw

**Affiliations:** a Sheffield Institute for Translational Neuroscience, Department of Neuroscience, School of Medicine and Biomedical Sciences, University of Sheffield, 385A Glossop Road, Sheffield S10 2HQ, UK; b Welsh School of Pharmacy, King Edward VII Avenue, Cardiff, CF10 3NB Wales, UK; c SimCyp, Blades Enterprise Centre, John Street, Sheffield, S2 4SU, UK

**Keywords:** ALS, amyotrophic lateral sclerosis, ARE, antioxidant response element, carboxy-H_2_DCFDA, 6-carboxy-2′,7′-dichlorodihydrofluorescein diacetate, CNS, central nervous system, DCF, dichlorofluorescein, MEFs, mouse embryonic fibroblasts, Nrf2, nuclear erythroid 2-related-factor 2, PBPK, physiologically based pharmacokinetic, Q-RTPCR, quantitative RT-PCR, Amyotrophic lateral sclerosis, motor neurone disease, Nrf2, preclinical pharmacology, neurodegeneration

## Abstract

Compelling evidence indicates that oxidative stress contributes to motor neuron injury in amyotrophic lateral sclerosis (ALS), but antioxidant therapies have not yet achieved therapeutic benefit in the clinic. The nuclear erythroid 2-related-factor 2 (Nrf2) transcription factor is a key regulator of an important neuroprotective response by driving the expression of multiple cytoprotective genes via its interaction with the antioxidant response element (ARE). Dysregulation of the Nrf2-ARE system has been identified in ALS models and human disease. Taking the Nrf2-ARE pathway as an attractive therapeutic target for neuroprotection in ALS, we aimed to identify CNS penetrating, small molecule activators of Nrf2-mediated transcription in a library of 2000 drugs and natural products. Compounds were screened extensively for Nrf2 activation, and antioxidant and neuroprotective properties *in vitro. S*[+]-Apomorphine, a receptor-inactive enantiomer of the clinically approved dopamine-receptor agonist (*R*[–]-apomorphine), was identified as a nontoxic Nrf2 activating molecule. *In vivo S*[+]-apomorphine demonstrated CNS penetrance, Nrf2 induction, and significant attenuation of motor dysfunction in the SOD1^G93A^ transgenic mouse model of ALS. *S*[+]-apomorphine also reduced pathological oxidative stress and improved survival following an oxidative insult in fibroblasts from ALS patients. This molecule emerges as a promising candidate for evaluation as a potential neuroprotective agent in ALS patients in the clinic.

## Introduction

Amyotrophic lateral sclerosis (ALS) is a devastating neurodegenerative disorder with rapid progression to death from neuromuscular respiratory failure in the majority of afflicted individuals. Only one therapeutic agent, riluzole, has been shown to ameliorate the disease course, with the modest effect of prolonging average survival by approximately 3 months. There is an urgent need for improved approaches to achieve neuroprotection in ALS.

Pathogenic mechanisms underlying ALS are not fully understood. Evidence from model systems, and from ALS patients, provides strong evidence for a role of oxidative stress in disease pathogenesis [1]. Oxidative stress has significant crosstalk with other potential mechanisms of neuronal injury, including mitochondrial dysfunction [2], excitotoxicity [3], protein aggregation [4], cytoskeletal dysfunction [5], glial cell activation [6], and the entry of TDP43 into stress granules [7,8]. It can feed into these mechanisms or be enhanced by them. This central role in pathogenesis is reflected in a meta-analysis of studies in the SOD1G93A transgenic mouse model of ALS where therapies targeting oxidative stress have been highlighted as demonstrating the greatest promise in slowing disease progression [9]. In ALS patients, there have been several trials of antioxidant therapies, but these have universally suffered from underpowered trial design or use of non-CNS penetrant or non-drug-like molecules [10,11]. Interestingly, a prospective epidemiological study of ∼1 million individuals showed that regular intake of the antioxidant vitamin E reduced relative risk of subsequent development of ALS to 0.38 [12]. Also riluzole, the only approved neuroprotective treatment for ALS, is an antiglutamatergic agent which also has antioxidant properties [13].

Despite this central role, therapeutic targeting of oxidative stress in ALS has failed to translate into clinical benefit for patients. This may be due in part to the lack of sufficiently potent antioxidants able to penetrate the central nervous system (CNS) [10]. An alternative novel approach for limiting oxidative stress in neurodegenerative disease is to promote activation of the transcription factor, NFE2-related factor 2 (Nrf2) [14,15].

Nrf2 is a Cap'n’Collar (CNC) basic region-leucine zipper (bZip) transcription factor with a transactivation domain in the N-terminal and a DNA binding region/leucine zipper structure in the C-terminal end [16]. It is negatively regulated by an actin binding cytosolic protein, Keap1 (Kelch-like ECH associated protein 1) [17], which acts as an adaptor and substrate binding domain for a Cul3-based E3 ubiquitin ligase. This complex associates with NRF2 via a “hinge and latch mechanism” [18,19] where a Keap1 homodimer binds two distinct motifs within the Neh2 domain of Nrf2, one of which forms a strong interaction (hinge) and the second a weak interaction (latch). When the Keap1–Nrf2 complex is exposed to oxidative stress or small molecule electrophiles, cysteine residues on Keap1 become oxidised, leading to formation of intermolecular disulfhide bonds and a conformational change [20,21]. This leads to dissociation of the weak interaction between Nrf2 and KEAP1, preventing ubiquitination of Nrf2 and effectively blocking the Keap1-based E3 ubiquitin ligase. Ultimately this increases net transfer of Nrf2 to the nucleus where it can drive expression of antioxidant enzymes [19,22,23]. The Keap1–Nrf2 complex thus constitutes a cytoplasmic sensor for oxidative stress and electrophilic agents [17].

Nrf2 drives expression of a battery of Phase II detoxification and antioxidant enzymes via its interaction with the antioxidant response element (ARE) [24,25]. When activated, this “programmed cell life” response is neuroprotective and conversely, attenuation of this pathway can enhance neuronal sensitivity to a range of neurotoxic challenges [15,26]. Dysregulation has been observed in ALS cellular models and confirmed in human tissue. We have previously demonstrated that both Nrf2 and multiple Nrf2 target genes showed decreased expression in a motor neuronal cell line expressing mutant (G93A) human SOD1 [27] and that the Nrf2 target, peroxiredoxin 3 (PRDX3), a mitochondrial antioxidant enzyme, is down-regulated in this cellular model and in human tissue from familial and sporadic ALS [28]. A reduction of Nrf2 transcripts and protein in spinal motor neurons and motor cortex from cases of sporadic ALS has also been described [29]. Astrocyte-specific expression of Nrf2 delayed the onset and extended survival in two mouse models of ALS [30]. This pathway thus represents an attractive therapeutic target due to its disease-specific dysregulation in ALS and the compelling evidence for a role of oxidative stress in disease progression. In addition, it is a well-defined target, amenable to activation by small molecules and activation of intrinsic cellular defence mechanisms may confer a more effective and enduring protection against oxidative stress than, for example, direct free radical scavenging.

A number of molecules have been described that activate the Nrf2-ARE pathway. These molecules tend to be natural products such as dietary flavanoids and cyclic sulfur-containing compounds [14]. These are not ideal therapeutic candidates, often with limited capacity to cross the blood brain barrier. The primary aim of the present program was to screen a commercial small molecule library containing both marketed drugs and natural products, with a view to identifying Nrf2-ARE activators which have the capacity to penetrate the CNS and be rapidly translated to clinical testing. We designed a comprehensive *in vitro* screening cascade culminating in assays of motor neuron survival in primary motor neuron/astrocyte cocultures. Of the 44 compounds initially identified, *S*[+]-apomorphine was selected for rigorous *in vivo* testing, including full pharmacokinetic analysis and its ability to activate the Nrf2-ARE pathway in the mammalian CNS. It was subsequently evaluated for neuroprotective potential in SOD1^G93A^ transgenic mice using objective and quantitative measures of disease progression. Finally, *S*[+]-apomorphine was evaluated in both sporadic and mutant SOD1-related ALS patient fibroblasts, which represent a peripherally accessible cellular model of human ALS. This is the first assessment of a CNS penetrating, Nrf2 activating small molecule which has followed the recommendations on preclinical studies in ALS mouse models [31] and which has evaluated the therapeutic potential in an *in vitro* model of human ALS.

## Results

### Reporter assay validation and library screening

**Fig. 1:**
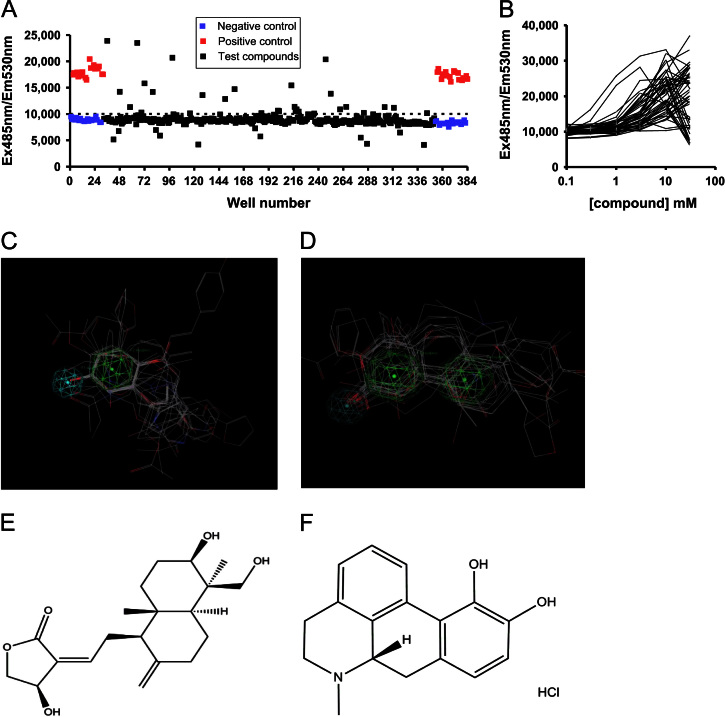
Compound library screening results. (A) Example Spectrum library screening data for one 384-well plate. GFP fluorescence versus well number. Wells 1–24 and 360–384 contain alternate positive (10 µM ebselen) and negative (vehicle) controls. Dotted line represents the average +3 SD of the negative controls and all compounds above this line are counted as “hits.” (B) Overlaid concentration response curves for all 44 hit compounds. Note that the profiles for many of the hit compounds follow a bell-shaped dose response curve, and have a narrow window of ARE induction. (C) Pharmacophore showing alignment of the 24 molecules with an EC50 of less than 10 µM in the primary screen (Supplementary Table 2) presented two common features: an aromatic/hydrophobic moiety and a hydrogen bond acceptor moiety. (D) When the 1321N1 astrocyte oxidative stress assay results (Supplementary Table 3) were used for constructing the pharmacophore with a 3 µM activity threshold, one additional common aromatic/hydrophobic feature was identified. Aromatic/hydrophobic feature in green, hydrogen bond acceptor feature in blue. This basic pharmacophore is consistent with known Nrf2 activators which may act by electrophilic attack of sulfhydryl groups on Keap1, the cytoplasmic Nrf2 regulator. The most potent compound from the primary screen, andrographolide (E), had an EC50 in this assay of 0.74 µM. One of the most potent and least toxic compounds in the secondary screens, *S*[+]-apomorphine is shown in (F).

In order to screen the 2000 compound Spectrum collection of known drugs and natural products, Nrf2-ARE reporter cell lines were generated in Chinese hamster ovary (CHO) cells and validated using known Nrf2 inducers *tert-*butyl hydroquinone (tBHQ) and the flavonoid epigallocatechin-3-gallate (EGCG, Supplementary Fig. 1a). The Spectrum library was screened twice, with compounds at 10 μM and simultaneous measurement of toxicity in both the reporter and the control cell lines. Example screening results for one 384-well plate are shown in Fig. 1A. A total of 44 compounds were identified as hits (positive response in both independent screens, no false positive response in the control cell line, and no evidence of toxicity) and assessed in full concentration response curves in the same assay (Fig. 1B). Most compounds showed toxicity at higher concentrations and a narrow window of Nrf2-ARE pathway activation, typical of molecules which activate this pathway.

Summary data for the hit ARE-inducing compounds emerging from this assay are shown in Supplementary Table 1 with a brief description of the known bioactivity of these compounds. The most potent ARE inducer was the natural product andrographolide, the only compound with a submicromolar EC_50_ (740 nM). This compound comes from the herb *Andrographis paniculata*, and is used widely in Chinese and Indian herbal medicine. There were 24 other natural products, and 19 synthetic small molecules or natural product derivatives and of these a total of 6 molecules were approved drugs, including *R*[–]-apomorphine, a dopamine agonist approved for the treatment of Parkinson’s disease.

### Pharmacophore for hit molecules

To rationalize the biological results, we attempted to identify a general pharmacophore for the hit compounds reported in Supplementary Table 2 using the Pharmacophore Elucidator implemented in MOE [Molecular Operating Environment (MOE 2007.09), Chemical Computing Group, Inc. Montreal, Quebec, Canada. http://www.chemcomp.com]. The pharmacophore that emerges is consistent with known Nrf2 activators which act by electrophilic attack of sulfhydryl groups on Keap1, the cytoplasmic Nrf2 regulator (Fig. 1C and D), although further experiments would be required to definitively prove this mode of action for the compounds identified in our assay.

### Effects of Nrf2-ARE-inducing hit compounds on oxidative stress induced by serum withdrawal in motor neuronal and astrocytic cells

To select the best candidates for further assessment we determined the potential of the 44 hit compounds to limit oxidative stress in CNS relevant cell types—a motor neuronal cell line (NSC34 cells) and rat (C6) and human (1321N1) astrocyte cell lines. The cell lines were pretreated with each of the hit compounds, at a range of concentrations for 24 h, to activate the Nrf2-ARE pathway. The compound was then removed and the cells subjected to a 6-h serum withdrawal to induce oxidative stress. Oxidative stress was measured using dichlorofluorescein (DCF) fluorescence and the degree of protection is shown as percentage reduction in DCF fluorescence for each of the three cell lines in Supplementary Table 2, with the IC_50,_ where available. Hit compounds were much less likely to show protective effects and more likely to increase oxidative stress in the motor neuronal cell line compared to the astrocyte cell line. Only 9/44 compounds reduced the oxidative stress DCF signal induced by serum withdrawal in NSC34 cells, whereas 17/44 increased the DCF signal (prooxidant). This compares to 29/44 and 32/44 protective compounds and 1/44 and 0/44 prooxidant compounds in the 1321N1 and C6 astrocytic cell lines, respectively. Since we were interested in identifying neuroprotective compounds, those compounds with a prooxidant effect in the NSC34 oxidative stress assay and known cytotoxic molecules were excluded, leaving 22 compounds

### Selection of likely CNS penetrant compounds

We next calculated the chemical/physical properties of the compounds, summarised in Supplementary Table 2. ALogP (log_10_ of the partition coefficient in octanol/water) and molecular polar surface area (mPSA) allow crude prediction of likely CNS penetrance. The Lipinski filter excludes compounds which are non-“drug-like.” Selection of compounds was refined based on the following criteria: AlogP>1, <4, mPSA<100. Applying these criteria to the remaining 22 molecules left 17 molecules for further investigation. These molecules are shown in Table 1 and are designated the “best hit” molecules.

### Nrf2-ARE-inducing activity of the best hit compounds in neuronal and astrocytic cell lines

**Fig. 2:**
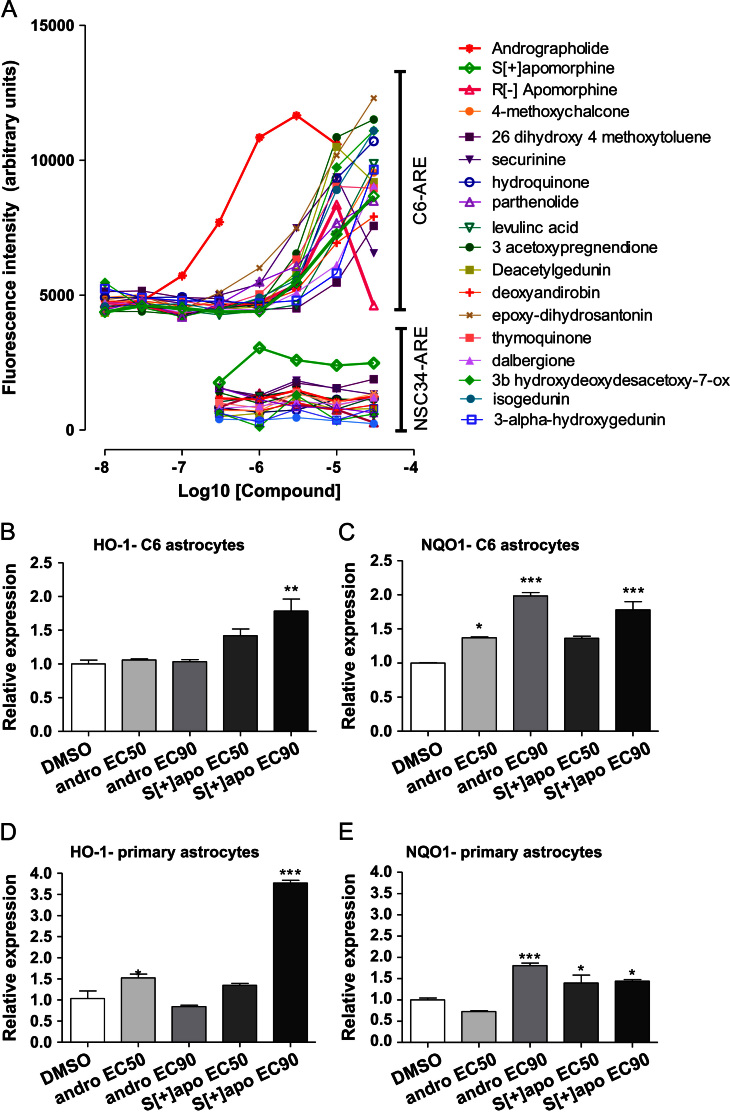
Hit compounds activate Nrf2-ARE directed transcription in C6 and primary mouse astrocytes. ARE reporter assay in C6 astrocyte cell line (C6-ARE) and NSC34 cell line (NSC34-ARE) for 17 best hit compounds and *S*[+]-apomorphine (A). Overall the response in C6-ARE is similar to that seen in the CHO-ARE cell line and both *R*[–] and *S*[+]-apomorphine induce the Nrf2-ARE pathway to a similar degree, although the *R* enantiomer shows toxicity at the highest dose (30 µM), suggesting that Nrf2 activation is unrelated to the dopamine agonist activity of *R*[–]-apomorphine. The response in the NSC34 cell line was substantially reduced or nonexistent for the majority of compounds, although *S*[+]-apomorphine shows some activation. Quantitative RT-PCR analysis for Nrf2-regulated genes in the rat C6 astrocyte cell line (B and C) and primary mouse astrocytes (D and E) following 24-h treatment with andrographolide (andro) and *S*[+]-apomorphine (*S*[+] apo) at EC50 and EC90 concentrations, as determined in C6–4xARE-TK reporter cells. Two key ARE genes, heme oxygenase 1 (HO-1, B and D) and NADPH quinone oxidoreductase-1 (NQO1, C and E), showed statistically significant changes in gene expression in both the rat C6 astrocytic cell line and the primary mouse astrocytes. Asterisks indicate significant difference from DMSO control by one-way ANOVA, *P<0.05, **P<0.01, ***P<0.001.

In order to determine whether the difference in protection in astrocytic and motor neuronal cell lines was due to differences in the degree of activation of the Nrf2-ARE pathway in these cell types, the Nrf2-ARE reporter construct was expressed in astrocytic (C6) and motor neuronal (NSC34) cell lines. The 17 best hit molecules were then screened in each cell line (Fig. 2A). We also screened the *S*[+] enantiomer of apomorphine. The *R*[–] enantiomer has dopamine agonist activity and is used in man, whereas the *S*[+] enantiomer lacks this dopaminergic activity [32]. In general, activation of the Nrf2-ARE pathway in the C6 cells was similar to that seen in the CHO cell line. The NSC34 reporter cell line showed minimal, if any, activation with the same set of concentration response curves, excepting *S*[+]-apomorphine which showed activation at 1 μM and above. This suggests that the underlying cause for greater protection against oxidative stress in astrocytic cell lines versus motor neuronal (NSC34) cells was due to a much more robust activation of the Nrf2-ARE pathway in astrocyte cell lines for most of the compounds. The *S*[+] enantiomer of apomorphine was equally potent in terms of Nrf2-ARE activation compared with the *R*[–] enantiomer in C6 cells, indicating that this activity is unrelated to dopamine receptor activation.

The key objective of this program of work was to identify molecules that could be fast-tracked for clinical testing in ALS, having been used previously in man. Of the 17 best hit molecules, two had a history of use in man as natural products (securinine, andrographolide) and one was a currently approved drug used for treatment of motor fluctuations in Parkinson’s disease (*R*[–]-apomorphine hydrochloride). Andrographolide and the *S*[+] enantiomer of apomorphine were selected for further assessment as the clinical experience for securinine is restricted to the former USSR and this compound is known to have convulsant properties [33]. The *S*[+] enantiomer of apomorphine was considered an attractive candidate for therapeutic assessment because it lacks the unwanted effects of the parent molecule (i.e., dopamine agonism, induction of emesis)

### Induction of ARE target gene expression and function in C6 cells and primary mouse astrocytes by andrographolide and S[+]-apomorphine

**Fig. 3:**
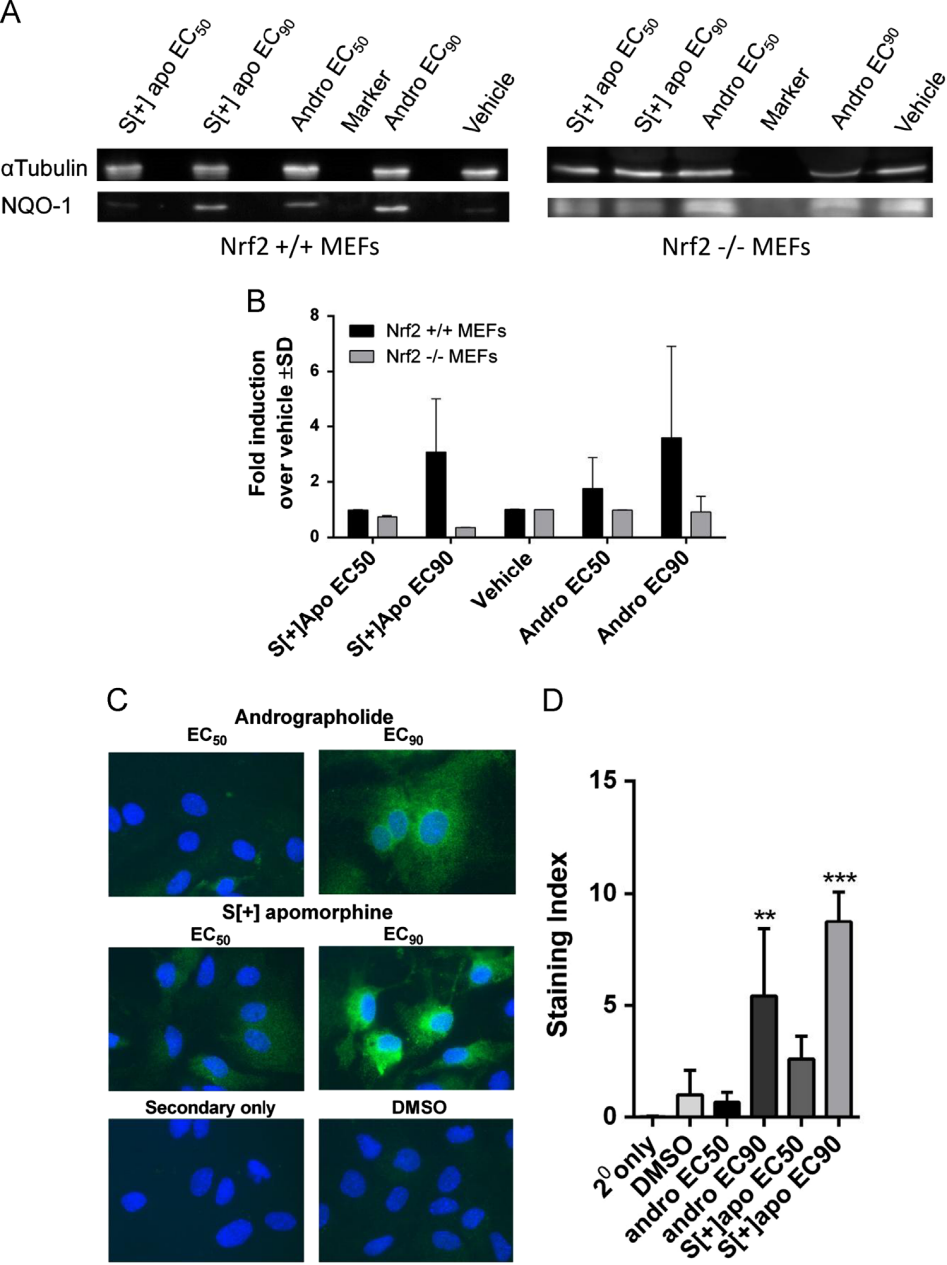
Andrographolide and *S*[+]-apomorphine increase antioxidant protein expression via activation of Nrf2 following treatment at EC_50_ and EC_90_ concentrations (as determined in C6–4xARE-TK reporter assay) for 24 h. (A) Western blotting for NQO-1 protein induction by *S*[+]-apomorphine and andrographolide in Nrf2 gene deleted mouse embryonic fibroblasts (Nrf2 −/− MEFs) and wild type (Nrf2 +/+ MEFs) controls. NQO-1 was probed in RIPA lysates of MEFs using a polyclonal antibody; α-tubulin was probed as a loading control. NQO-1 gave a calculated molecular weight of 27 kDa and alpha-tubulin 54 kDa. The overall level of NQO-1 expression was lower generally in Nrf2 −/− MEFs, necessitating longer exposures for detection. (B) Densitometry analyses from two independent Western blotting experiments showing fold induction of NQO-1 over vehicle control, both *S*[+]-apomorphine and andrographolide show *a*>3-fold induction of NQO-1 at EC_90_ concentrations in wild-type MEFs, whereas there is no induction in Nrf2−/− MEFs. (C) Immunofluorescence staining for heme oxygenase 1 (HO-1) in primary mouse astrocytes. (D) Area and staining intensity were quantified using Image J and used to calculate a staining index. A significant increase in HO-1 staining was observed following treatment with EC90 concentrations of *S*[+]-apomorphine and andrographolide compared to DMSO control (one-way ANOVA with Bonferroni post tests, **P<0.01, *P<0.001).

To confirm that these “lead inducers” were able to activate the Nrf2-ARE pathway, quantitative RT-PCR (Q-RTPCR) for Nrf2 target genes NADPH quinone oxidoreductase 1 (NQO1) and heme oxygenase-1 (HO-1) was performed in C6 cells (Fig. 2B and C) and primary mouse astrocytes (Fig. 2D and E) following treatment with andrographolide and *S*(+)-apomorphine at EC_50_ and EC_90_ concentrations (as determined in the Nrf2-ARE reporter assay in C6 cells) and gene induction was observed in most conditions. Further experiments were conducted in Nrf2 −/− mouse embryonic fibroblasts (MEFs) to confirm that Nrf2 was essential to the response (Fig. 3A and B). In wild-type MEFs, both *S*[+]-apomorphine and andrographolide enhanced NQO1 protein expression by 3.1-fold and 3.6-fold, respectively, when dosed at EC_90_ concentrations. This compared to a similar level of expression in Nrf2−/− MEFs for andrographolide at EC_90_ concentrations (0.92-fold change versus vehicle) as opposed to a reduction in expression for *S*[+]-apomorphine at EC_90_ concentrations (0.36-fold change versus vehicle). Induction of HO-1 at the RNA level was associated with a dose-dependent enhancement of protein expression as demonstrated by immunofluorescence staining (Fig. 3C and D).

**Fig. 4:**
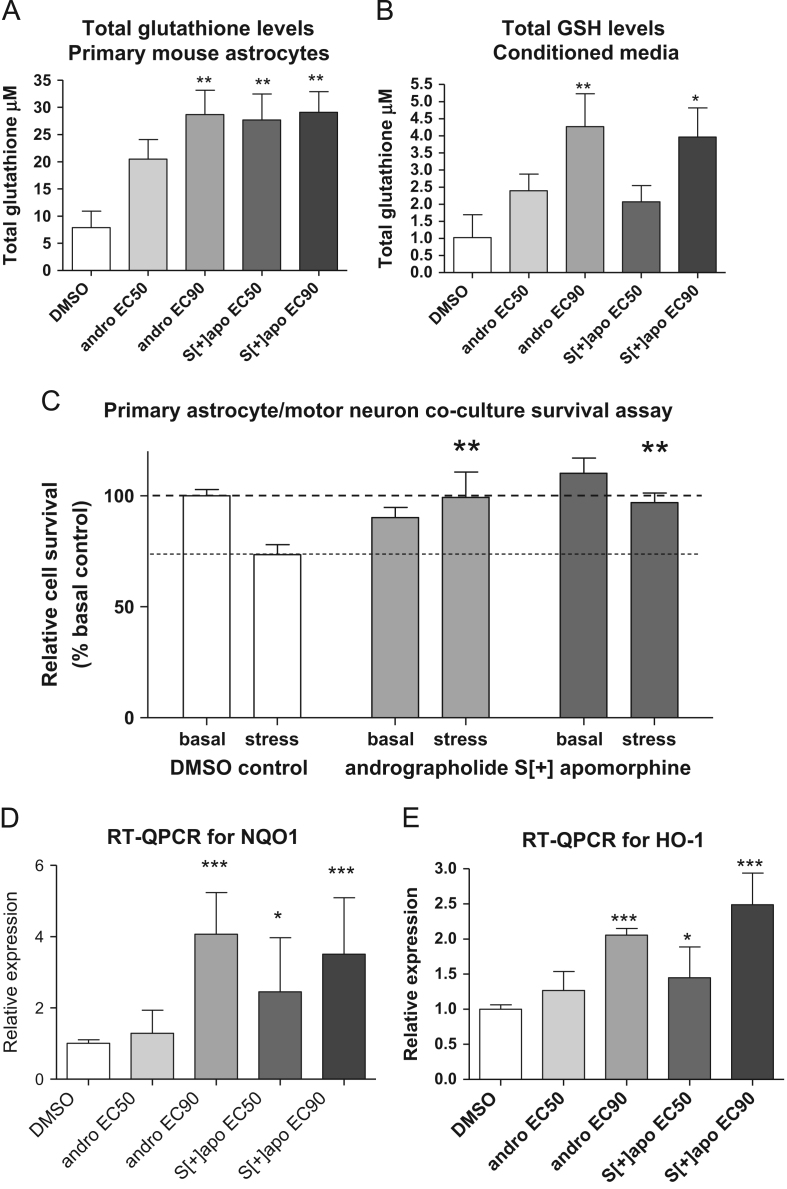
Andrographolide and *S*[+]-apomorphine enhance glutathione production and antioxidant gene transcription in primary mouse astrocytes and protect motor neurons (MN) in coculture with astrocytes from oxidative stress (menadione)-induced cell death. (A) Total glutathione levels in primary mouse astrocytes and (B) in conditioned media collected from primary mouse astrocytes. Total glutathione levels were measured following 24-h pretreatment with Nrf2 inducers. Data are average +/– SEM of four independent experiments. (C) Nrf2 inducers protect MN in primary mouse astrocyte/MN cocultures from menadione stress. Cocultures were pretreated for 24 h with *S*[+]-apomorphine and andrographolide at their EC50 and EC90 concentrations, respectively. The cocultures were then challenged for 6 h with 10 µM menadione to induce oxidative stress. In DMSO control cells an approximately 25% reduction in MN number was observed which was not seen in cultures treated with either drug. (D) Quantitative RT-PCR analysis for the Nrf2-regulated genes NQO1 and (E) heme oxygenase 1 (HO-1), in primary mouse astrocytes from SOD1G93A transgenic mice. *S*[+]-Apomorphine (*S*[+]-apo) and andrographolide (andro) were at EC50 and EC90 concentrations, as determined in rat C6 4xARE-TK reporter cells in all experiments. Asterisks indicate significant difference from DMSO control by one-way ANOVA with Bonferroni multiple comparison test *P<0.05, **P<0.01, ***P<0.001.

An important functional effect of this enhanced antioxidant capacity was demonstrated by measuring total glutathione levels under the same conditions in both primary astrocytes and in the media collected from treated astrocytes (Fig. 4A and B). Glutathione levels were elevated within both the astrocytes and the media—highlighting one mechanism by which Nrf2-responsive astrocytes may protect neighbouring, less ARE responsive, MNs from oxidative challenge.

### Nrf2 Inducers protect motor neurons from oxidative stress in primary mouse astrocyte/motor neuron cocultures

Cocultures consisting of primary mouse motor neurons (MNs) on an astrocyte feeder layer were exposed to an oxidative insult following pretreatment with either andrographolide or [*S*+]-apomorphine (Fig. 4C). The cocultures were then challenged for 6 h with 10 µM menadione to induce oxidative stress and MNs stained and counted. In DMSO control cultures an approximately 25% reduction in MN number was observed. In cocultures treated with either andrographolide or [*S*^+^]-apomorphine, significant neuroprotective effects were observed, expressed as a preservation of MN numbers in the presence of menadione induced oxidative stress (Fig. 4C).

### Andrographolide and S[+]-apomorphine induce HO-1 and NQO1 in G93A SOD1 expressing astrocytes

Previous work had demonstrated an attenuated Nrf2 response in motor neuronal cell lines expressing mutant SOD1, and in postmortem tissue from human cases of SOD1-related ALS [27,29]. It was therefore important to determine whether our lead inducers could still activate the Nrf2 pathway in astrocytes expressing G93A mutant SOD1. Q-RTPCR was performed and demonstrated a significant increase in transcripts for NQO1 and HO1 following a 24-h pretreatment with andrographolide and *S*[+]-apomorphine at their EC_90_ concentrations and for andrographolide at its EC_50_ concentration (Fig. 4D and E).

### Preclinical efficacy of S[+]-apomorphine in the SOD1^G93A^ mouse model of ALS

**Fig. 5:**
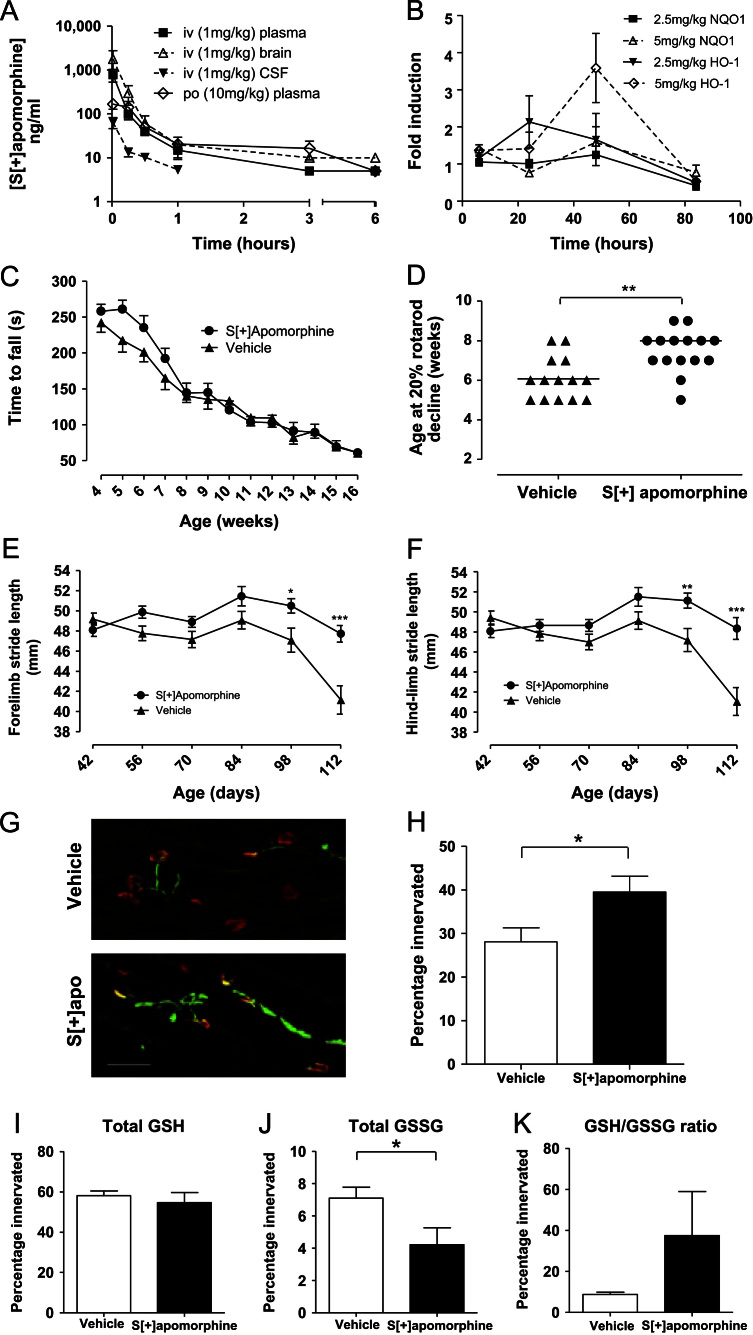
In vivo profiling of *S*[+]-apomorphine. (A) Pharmacokinetic (PK) analysis was conducted in normal C57Bl/6 mice following both intravenous (iv, 1 mg/kg) and oral (po, 10 mg/kg, *n*=3 per time point po and 4 per time point iv). For calculated PK parameters see Supplementary Table 4. (B) Measurement of HO-1 and NQO1 gene expression in whole spinal cord at 6, 24, 48, and 84 h postdosing with 5 mg/kg *S*[+]-apomorphine via the sc route. (C–H) Female SOD1G93A transgenic mice were dosed from Day 21 until end-stage (*n*=13–15 per group), daily with 5 mg/kg *S*[+]-apomorphine sc. A pronounced decline in rotarod performance is observed in our model from p40 in SOD1G93Acontrol mice (C); this initial decline was significantly delayed by treatment with *S*[+]-apomorphine as assessed by the time taken to reach a 20% decline in rotarod performance (D, median 6 weeks for vehicle-dosed mice, 8 weeks for *S*[+]-apomorphine-dosed mice, *P*=0.0033 Mann-Whitney *U* test). At later stages of disease, gait analysis showed a significant delay in decline of both fore-limb (E) and hind-limb (F) stride length for *S*[+]-apomorphine-treated mice (two-way ANOVA with Bonferroni post test). (G) Immunofluorescence staining for motor end plates (alpha-Bungartoxin, red) and presynaptic processes (combined synaptophysin and neurofilament, green) in gastrocnemius muscle of vehicle and *S*[+]-apomorphine-treated mice at 60 days of age showed a significant protection against loss of innervation (H, Student's *t* test, *n*=3 per group). (I) Measurement of oxidised and (R) reduced glutathione levels in spinal cord at 60 days showed a significant reduction in oxidised glutathione levels and a higher GSH/GSSG ratio (K) in *S*[+]-apomorphine-treated mice (*n*=7 per group). *P<0.05, **P<0.01, ***P<0.001.

CNS bioavailability of both *R*[–] and *S*[+]-apomorphine has been previously described [34]. For this reason we selected *S*[+]-apomorphine for proof of concept studies *in vivo* in the SOD1^G93A^ mouse model of ALS. A rational approach, mirroring clinical phase I, II, and III treatment trials, was taken in this chronic and aggressive preclinical model. First, pharmacokinetic (PK) analysis was conducted (Fig. 5A) in normal C57Bl/6 mice following intravenous dosing at 1 mg/kg with assessment of drug levels in plasma, brain, and cerebrospinal fluid (CSF) by LC/MS-MS. To assess oral bioavailability, *S*[+]-apomorphine was also dosed orally at 10 mg/kg and the plasma time curve established (see Supplementary Table 3 for calculated pharmacokinetic parameters). Plasma half-life was short (∼11 min), although similar to that described previously for *R*[+]-apomorphine. Brain uptake was calculated as the area under the curve to infinity (AUC_infinity_) in brain tissue divided by the AUC_infinity_ in plasma. A value of 228% was calculated, indicating CNS penetration of the parent compound. Since subcutaneous (sc) bioavailability is almost 100% [35], the sc route was selected and dose levels of 2.5 and 5 mg/kg were tested for ability to induce Nrf2-regulated gene expression in the spinal cord (Fig. 5B). Gene expression of both HO-1 and NQO1 was assessed by Q-RTPCR in whole spinal cord at 6, 24, and 48 h postdosing and 5 mg/kg gave a 3.5-fold HO-1 gene induction at 48 h. Physiologically based pharmacokinetic (PBPK) modeling was also used to determine the likely CNS concentrations following these subcutaneous doses of 2.5 and 5 mg/kg (Supplementary Fig. 2). The PBPK model was able to describe the observed plasma concentrations after intravenous (iv) and oral dosing and the total brain concentrations observed after iv dosing (Supplementary Figs. 2a–c). Simulations of the exposure following sc administration at 2.5 and 5 mg/kg predict that maximal plasma concentrations at the lower dose were in the range of 1.2–1.9 μM (Supplementary Fig. 2d) and at the higher dose were in the range of 2.4–3.8 μM (Supplementary Fig. 2e). Concentrations in brain were in the range of 2.7–4.3 and 5.4–8.5 μM at doses of 2.5 and 5 mg/kg, respectively (Supplementary Figs. 2f and g), possibly explaining the differences in gene induction seen with these two doses.

As the drug was able to access the CNS and induce gene expression, a full pharmacology study in SOD1^G93A^ transgenic mice was initiated (*n*=15/group) dosed from Day 21 until end-stage, daily with 5 mg/kg *S*[+]-apomorphine sc. The rotarod test (Fig. 5C and D) was performed weekly and semiautomated gait analysis using the Catwalk system (Fig. 5E and F) was performed every 2 weeks from Postnatal Day 42 (p42) to p112. A pronounced decline in rotarod performance is always observed in our model from p40 in mSOD1^G93A^ mice [36]. This initial decline of motor function was significantly delayed by treatment with *S*[+]-apomorphine (Fig. 5C) as assessed by the time taken to reach a 20% decline in rotarod performance (Fig. 5D, median 6 weeks for vehicle-dosed mice, 8 weeks for *S*[+]-apomorphine-dosed mice, *P*=0.0033, Mann-Whitney *U* test). At later stages of disease, gait analysis showed a significant delay in decline of both fore-limb (Fig. 5E) and hind-limb (Fig. 5F) stride length for *S*[+]-apomorphine-treated mice (two-way ANOVA with Bonferroni post test). A separate group of mice was assessed for the degree of gastrocnemius muscle innervation (Fig. 5G and H) and reduced and oxidised glutathione levels at 60 days of age (Fig. 5I, J, and K). A significant increase in muscle innervation was observed with *S*[+]-apomorphine treatment (Figs. 5H, 39.5% versus 28.1%, *P*=0.0340, Student’s *t* test) and a significant reduction in oxidised glutathione levels in spinal cord (Figs. 5J, 4.2 μM±1.0 versus 7.1 μM±0.67, *P*=0.038, Student’s *t* test). Despite significant slowing of disease progression, we did not observe a significant improvement in survival (measured by failure of the righting response within 10 s) or motor neuron numbers at end-stage in the *S*[+]-apomorphine-treated group.

### Fibroblasts from ALS patients display elevated levels of oxidative stress which can be attenuated with S[+]-apomorphine

**Fig. 6:**
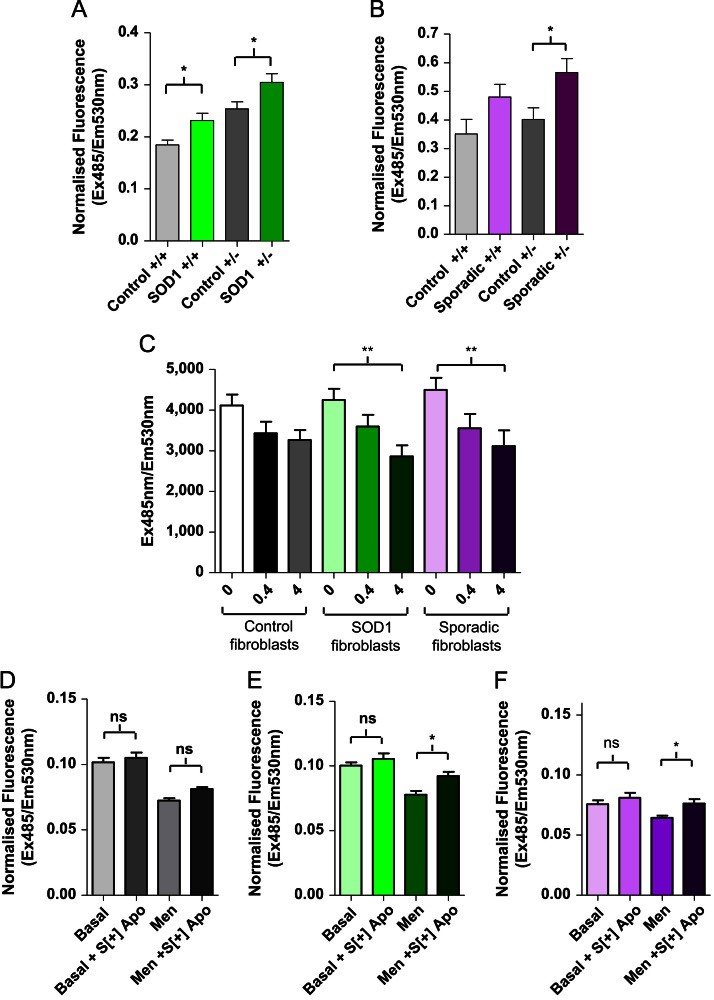
Oxidative stress and cell death assays in fibroblasts from ALS and control patients. Fibroblasts from SOD-1-related and sporadic ALS patients display an elevated level of oxidative stress which is significantly attenuated by treatment with *S*[+]-apomorphine. (A) Effect of the I113T SOD1 (*n*=3 cases) mutation on oxidative stress, measured using dichlorofluorescein (DCF) fluorescence in human fibroblasts from three patients under basal conditions in media with (+/+) and without (+/–) glutamine compared to control patients (*n*=3 cases) under the same conditions. (B) Oxidative stress in fibroblasts of sporadic ALS patients (*n*=2 cases) under the same conditions compared to control patients (*n*=3 cases). (C) *S*[+]-Apomorphine at a concentration of 4 µM significantly reduces oxidative stress, measured as DCF fluorescence, in sporadic and SOD1-related ALS patient fibroblasts and to a greater extent than the reduction seen in control fibroblasts which is nonsignificant. Fibroblasts from a control (D), an I113T SOD1 patient (E), and a sporadic ALS patient (F) were treated with 4 µM *S*[+]-apomorphine (*S*[+] Apo) with and without exposure to menadione (Men) to induce cell death measured by MTT assay (see methods). *S*[+]-Apomorphine significantly attenuated cell death in both sporadic and I113T SOD1 ALS cases but not in the control case. Data analysed by two-way ANOVA with Bonferonni post tests, *P<0.05, **P<0.01.

In order to establish the relevance of *S*[+]-apomorphine as a potential therapy in human ALS, we isolated primary fibroblasts from both sporadic and I113T mutant SOD1-related ALS patients. These fibroblasts showed an elevated level of oxidative stress compared to control fibroblasts (Fig. 6A and B) and represent a useful model for screening candidate antioxidants. When *S*[+]-apomorphine was tested at 0.4 and 4 µM, a significant reduction in basal oxidative stress was observed in both sets of the patient fibroblasts but not in the control fibroblasts at 4 µM (approximate EC_50_ concentration, Fig. 6C). *S*[+]-Apomorphine was also able to protect against menadione-induced cell death in ALS patient fibroblasts (Fig. 6D and E).

## Discussion

The objective of this program of work was to identify potent Nrf2-inducing molecules with the potential to penetrate the CNS and a previous history of safe use in man, enhancing the potential for rapid clinical development. Screening a library of 2000 drugs and natural products led to the identification of 44 molecules, which had the capacity to activate the Nrf2 pathway in a reporter cell line.

The approach used was particularly suited to the Nrf2 pathway because many small molecule compounds (natural and synthetic) are known to activate this pathway, although such activation is often linked with toxicity. Of the compounds identified, one natural product (andrographolide) and the receptor inactive enantiomer of a clinically approved dopamine agonist (*S*[+]-apomorphine) were selected for further investigation.

Andrographolide is the principal active component in extracts of the herb *Andrographis paniculata* [37] which is widely used in Indian herbal medicine as an anti-infective, anti-inflammatory and hepatoprotective agent. Andrographolide, a diterpene lactone, has not previously been shown to directly activate the Nrf2-ARE pathway, although its antioxidant capability has been recognised [38,39]. Clearly, the activation of the Nrf2-ARE pathway that we have identified provides a defined mechanism by which andrographolide can mediate these antioxidant effects. However, there are no data on the distribution of andrographolide in the CNS as far as we are aware and andrographolide appears to be a substrate for P-glycoprotein [40], one of the major efflux transporters in brain endothelium [41] which is predicted to inhibit efficient delivery of andrographolide to the CNS.

In contrast, apomorphine has established CNS activity and is known to partition to the CNS. *R*[-]-Apomorphine is a nonselective dopamine D1/D2 agonist. It shows more potent D2-like (D_2_, D_3_, and D_4_) activity, reflected in high binding affinity for D4 receptors (*K*_i_=4.4 nM), and more limited D_1_-like (D_1_ and D_5_) activity, with low binding affinity for D1 receptors (*K*_i_ 370 nM) [42]. Originally used as an emetic, it is administered parenterally for the rescue of “off” episodes in Parkinson’s disease (PD) [43]. Apomorphine has a well-described pharmacokinetic profile in man [35,44,45] and in rats, demonstrating significant accumulation in the CNS [46].

Although *R*[–]-apomorphine is a potent dopamine agonist, the *S*[+] enantiomer is not [47] and evaluation in *in vivo* models of dopamine receptor activation failed to evoke a response at doses up to 25 times those required with *R*[–]-apomorphine [32]. In addition, *S*[+]-apomorphine failed to induce emesis in dogs at doses 10-fold greater than those required with *R*[–]-apomorphine [32]. *S*[+]-Apomorphine can antagonise the effects of *R*[–]-apomorphine and although it has some D1/2 antagonist activity [47] this is very unlikely to explain the improvement in parameters of motor function seen in our mouse model since it is rapidly cleared (*T*_1/2_ 11 min, Supplementary Table 4) and D1 and D2 dopamine antagonists are known to inhibit rather than enhance locomotor activity [48,49].

Apomorphine has recognised antioxidant activities. It was able to inhibit Fenton reaction-mediated lipid peroxidation in rat brain mitochondrial fractions—attributed to an ability to directly scavenge ROS [50]; protected rat PC12 cells from oxidative damage at low micromolar concentrations [51] and reduced MPTP-induced dopaminergic toxicity in mice [52]. *R*[–]-Apomorphine was shown to protect SH-SY5Y cells from 6-hydroxydopamine-induced cell death via activation of the Nrf2-ARE pathway [53].

We have now demonstrated that both the *S*[+] and the *R*[–] enantiomers of apomorphine are able to activate the Nrf2-ARE pathway with similar potency and this opens up the interesting possibility of using the *S*[+] form as a tool to activate the Nrf2-ARE pathway *in vivo*. The advantages of this approach are obvious; the lack of dopamine agonism and emesis with the *S*[+] form will enable dosing in a higher range with a greater safety margin in clinical studies, yet the desirable properties of CNS partitioning and Nrf2-ARE activation are maintained.

The protective effects of Nrf2 on motor neurons could be via both indirect and direct mechanisms. We have shown that induction of Nrf2 in astrocytes by *S*[+]-apomorphine promotes antioxidant defence via enzyme induction and upregulation of intracellular and extracellular secreted glutathione levels. This enhanced glutathione production could in turn protect neighbouring motor neurons from oxidative insults in an indirect manner. *S*[+]-Apomorphine was the only compound tested which was able to activate the Nrf2 pathway in a motor neuronal cell line raising the possibility that direct activation of Nrf2 signaling in motor neurons could also enhance antioxidant defences.

The pharmacokinetic and pharmacodynamic profile of *S*[+]-apomorphine demonstrates long-term induction of the Nrf2-mediated response following a relatively short exposure, which translates to a significant beneficial effect on motor function measured using quantitative and objective measures of disease progression in a commonly used mouse model of ALS. However we cannot exclude the possibility that the mechanism of action *in vivo* may involve other pathways than enhancement of antioxidant defences. Nrf2 can activate transcription of a wide variety of genes and gene families including those associated with xenobiotic metabolism, protein stress response, and inflammation [54,55], all of which are implicated in mutant SOD1-mediated neurotoxicity [1]. In addition the precise set of genes activated varies with different activators [55]. The transcriptional response following *S*[+]-apomorphine treatment is currently under investigation in our laboratory.

Although no improvement in mouse survival was observed in our model, improvements in measures of disease progression are given equal weight to measures of improved survival in the recent guidelines [31] and this is now becoming more accepted in the field [56]. These parameters also relate to well-defined, early disease processes [36] shown to be active in human ALS [57] and therefore have the potential to translate into significant outcome benefits in the more gradually progressive human disease. Furthermore, the use of survival as an endpoint in the SOD1^G93A^ mouse model has so far failed to deliver clinically translatable effects [9] and few of the positive effects on survival described in this aggressive model have been well replicated [58], likely due to poor study design in the mixed genetic background mice (SJLBL6) used in the majority of studies [59]. Indeed there have been two previous studies using Nrf2-activating molecules in the SOD1^G93A^ mouse model [60,61], but neither of these studies has followed the recommended study design [59] for the mixed SJLBL6 background and the effects reported could well be due to confounding factors (effect of litter, changes in transgene copy number).

As proof of concept that activation of Nrf2 might have a beneficial effect in human ALS, we were able to demonstrate that *S*[+]-apomorphine reduced pathologically elevated levels of oxidative stress in fibroblasts from patients with both mutant SOD1-related and sporadic ALS, and could reduce oxidative stress-induced cell death in this human cell model. The combination of efficacy in a clinically translatable readout (motor function in SOD1^G93A^ mice) and reduction in oxidative stress observed in fibroblasts from ALS patients gives confidence in progressing *S*[+]-apomorphine toward the clinic. This is the first time that a clinically acceptable, CNS penetrant molecule has been shown to activate the Nrf2-ARE pathway *in vivo* and ameliorate disease measures in both mouse and patient fibroblast models of ALS.

The next stages in this program entail development of biomarkers of drug action to enable proof of mechanism studies in man, an approach already taken in oncology in so-called “Phase 0” clinical studies [62]. We believe this represents a rational and expeditious approach to bring to the clinic new therapeutic approaches for ALS and potentially for other CNS disorders associated with oxidative stress.

## Materials and methods

### Cell culture

Chinese hamster ovary (CHO), NSC34 mouse motor neuronal cells [63] C6 (rat), and 1321N1 (human) astrocytic lines were routinely maintained in DMEM supplemented with 10% FBS and penicillin/streptomycin. The ARE-TK-GFP and TK-GFP reporter constructs were a kind gift from William E. Fahl McArdle Laboratory for Cancer Research, University of Wisconsin. The TK-EGFP reporter construct consists of a 123-bp thymidine kinase promoter inserted in the multiple cloning site of pEGFP (Clontech) and the ARE-TK-EGFP also contains four repeats of a 41-bp GST ARE motif (TAGCTTGGAAATGACATTGCTAATCGTGACAAAGCAACTTT) 3′ to the TK promoter [64]. These plasmids were transfected into CHO, C6, and NSC34 cell lines using Lipofectamine 2000 (Invitrogen) and following 10–14 days of selection in 0.5 mg/ml G418 they were expanded and selected for basal eGFP expression using fluorescence-activated cell sorting (BD, FACSAria) with two sequential cell sorts for each cell line. These mixed populations of stable transfectants with basal eGFP expression were used in subsequent assays and designated 4xARE-TK-GFP for the ARE-containing line and TK-GFP for the control cell line. The NSC34 cell lines stably expressing G93A mutant hSOD1 have been described previously [65]. In brief, NSC34 cells were transfected with G93A mutant SOD1 and stably transfected single cell clones were isolated by selection in 250 μg/ml G418 and cloning by limiting dilution. Mouse embryonic fibroblasts were generated and maintained as described previously [66].

### ARE reporter assay—Spectrum library screen validation

In order to screen the Spectrum library of 2000 small molecule drugs and natural products the TK-GFP CHO ARE reporter cell line was subjected to a *Z*′ score assay [67] in a 384-well plate (Greiner Bio-one, μClear, black) using a range of plating densities (5–20×10^4^/well plated 24 h prior to assay) and different media. Alternate wells were incubated with 10 μM Ebselen and vehicle (0.1% DMSO) for 24 h followed by replacement of media with PBS containing 0.3 μM ethidium homodimer-1 (EthD1, Molecular Probes, Paisley, UK). This concentration of Ebselen represents an approximate EC_90_ for this drug. GFP fluorescence (ARE induction) was then measured at Ex_485 nm_/Em_530 nm_ using a Fusion universal plate reader (Packard Bioscience). The *Z*′ score was calculated as follows

$$Z\prime =\frac{1-({\mathrm{3SD}}^{+}+{\mathrm{3SD}}^{-})}{\left({\mathrm{Ave}}^{+}–{\mathrm{Ave}}^{-}\right)}$$

where *SD*^+^ = standard deviation of positive control wells; *SD*^−^ = standard deviation of negative control wells; Ave^+^ = average fluorescence reading of positive control wells; Ave^−^ = average fluorescence reading of negative control wells.

Signal to noise (*S*/*N*= Ave^+^/SD^+^) and signal to background (*S*/*B* = Ave^+^/Ave^−^) ratios were also determined for the different assay conditions. Acceptable *Z*′ scores were >0.5.

For the library screen, cells were plated at a density of 20×10^4^ in normal DMEM media containing 10% FBS on Day –1 and on Day 0, cells were incubated for 24 h with drug in serum-free media. Media was removed by hand and replaced (1 compound/well) with the Spectrum library diluted to 10 μM in 0.1% DMSO using a Q-bot liquid handling system (Genetix, New Milton, UK). The media were removed after 24 h and replaced with the same volume of PBS containing 0.3 μM EthD1. GFP fluorescence (ARE induction, Ex_485 nm_/Em_530 nm_) and Eth D1 fluorescence (toxicity Ex_530 nm_/Em_645 nm_) were then measured. The TK-GFP CHO ARE cell line was screened twice in a single point assay and the control TK-GFP CHO cell line was screened once to eliminate false positives.

### ARE reporter assay—Determination of EC_50_

Reporter assays were run as for the library screen with a concentration curve ranging from 0.01 to 100 μM drug in triplicate in FCS-free DMEM for 24 h. Nonlinear regression was used to fit a sigmoidal dose–response curve on a semi-Log plot to calculate the EC_50_ using GraphPad Prism (GraphPad Software). The reporter assay was performed similarly in C6 and 1321N1 astrocyte cell lines stably transfected with the 4xARE-TK-GFP and TK-GFP constructs except that EthD1 was added directly in the media and read prior to washing the cells and reading the GFP signal.

### Oxidative stress assay

The NSC34, C6, and 1321N1 cells were plated in 96-well tissue culture plates to achieve 30% confluency and incubated with drug in triplicate wells as a 9 point titration (100 µM to 10 nM) for 24 h. Cell density was observed to ensure that no significant toxicity or growth inhibition occurred. Media were then replaced with serum-free, phenol-free media for 5 h. Carboxy-H_2_DCFDA (6-carboxy-2′,7′-dichlorodihydrofluorescein diacetate, di(acetoxymethyl ester), and EthD1 were added to the cells to final concentrations of 5 and 0.3 μM, respectively. Carboxy-H_2_DCFDA and EthD1 fluorescence was read at Ex_485 nm_/Em_530 nm_ and Ex_530 nm_/Em_645 nm_, respectively, after 1 h. Cell survival assay was then performed on the cells as protection is measured as percentage reduction in carboxy-H_2_DCFDA signal; therefore data points were excluded where a decrease in cell number was measured.

### Cell viability assay

The method used was essentially as described previously [65]. Briefly methylthiazolyldiphenyl-tetrazolium bromide (MTT) was added to the cells and a blank well to a final concentration 0.5 mg/ml and incubated at 37 °C for 1–3 h depending on the cell line used. Cells and reaction product were solubilised in 20% SDS/50% DMF for 1 h with shaking at room temperature before reading the absorbance at 595 nm.

### Pharmacophore development and modeling

All molecular modeling studies were performed on a MacPro dual 2.66GHz Xeon running Ubuntu 8. Compounds were built with Molecular Operating Environment (MOE) 2007_09 and minimized using the MMFF94x force field until a RMSD gradient of 0.05 kcal mol^−1^ Å^−1^ was reached. The Pharmacophore Elucidator implemented in MOE was used to generate a pharmacophore, performing a full conformational search on the compounds selected.

### Primary mouse motor neuron/astrocyte cocultures

Mouse glial cultures were established based on published methods [68,69]. Primary spinal cord motor neurons (MNs) were cultured from E13.5 C57Bl/6 mouse embryos as described previously [70]. Cocultures were allowed to establish for 2 weeks and neuroprotection assays performed. Cocultures were exposed to drug or vehicle for 24 h followed by a 6-h treatment with menadione (10 μM) to induce oxidative stress or with no stress to determine the effect of drug alone. Following stress treatment, coverslips were washed 3 times, fixed, and permeabilised, and MNs immunostained with SMI32 (Covance, UK). Total MNs were counted by fluorescence microscopy in a 1.5 cm^2^ area per coverslip. A minimum of three repeats in triplicate were performed per condition. Both vehicle and drug treatments were counted with and without stress treatment, and results were analysed by two-way ANOVA using Bonferroni post tests.

### Western blot analysis

Cells were isolated and lysed in a RIPA buffer containing protease inhibitor cocktail (Roche), quantified for protein content by Bradford assay, and loaded 30 μg per sample. Samples were run on a 12.5% acrylamide gel under reducing conditions with a prestained marker and transferred to polyvinylidene fluoride membrane and blocked in 5% milk. Mouse anti-alpha-tubulin, clone DM1A (Sigma), and rabbit anti-NQO1 (kind gift from Prof. John Hayes, University of Dundee) were used at 1 in 1000 dilution in Tris-buffered saline containing Tween. Polyclonal goat anti-mouse (Abcam-Ab97040) and polyclonal goat anti-rabbit (Dako Cat. No. P0448), horseradish peroxidase-conjugated secondary antibodies were used for detection of relevant primary antibodies. Bands were visualised by enhanced chemiluminescence reagents (Geneflow EZ-ECL kit). Relative band intensities were captured, using subsaturation, and quantified using a Gbox iChem XT digital imaging system (Syngene).

### Total glutathione assay

Primary astrocytes were grown to confluency in 24-well plates and then treated with drug (or 0.05% DMSO vehicle) in phenol red-free DMEM containing 10% FBS and penicillin/streptomycin for 24 h. Conditioned medium was collected and astrocytes were then washed in ice-cold PBS before addition of 250 μl/well of sulphosalicylic acid (SSA, 5% (w/v)). Plates were frozen at −80 °C and thawed at 37 °C, twice, and then incubated at 4 °C for 15 min. The supernatant was removed and centrifuged at 13,000 *g* for 5 min. Conditioned medium samples were incubated at 80 °C for 15 min and then centrifuged at 13,000 *g* for 5 min. Samples were either used immediately or stored at −80 °C. Reaction mixture (150 μl/well; 100 mM potassium phosphate buffer, pH 7.0, 1 mM EDTA, 6 Units/ml glutathione reductase, 1.5 mg/ml 5,5′-dithiobis(2-nitrobenzoic acid)) was added to 10 μl of each sample or glutathione standard (0–50 μM reduced glutathione) in a 96-well plate and incubated at room temperature for 5 min before addition of 50 μl/well of NADPH solution (0.16 mg/ml). *A*_412nm_ was measured every minute for 15 min and the total glutathione concentration (GSH + GSSG) was calculated from initial rates. Samples were tested in triplicate.

### Quantitative RT-PCR

Nrf2 Target gene expression was determined using Q-RTPCR as described previously [28] using the following primers: Ho-1 forward primer, cac ttc gtc aga ggc ctg cta; Ho-1 reverse primer, gcg gtg tct ggg atg agc ta; Nqo1 forward primer, cgc ctg agc cca gat att gt; Nqo1 reverse primer, act gca atg gga act gaa ata tca; GAPDH forward, gaa acc tgc caa gta tga tga cat; GAPDH reverse, ggt cct cag tgt agc cca aga t.

### In silico analysis

In order to select drug-like molecules for further screening, Pipeline Pilot (SciTegic, London, UK) was used for *in silico* analysis. The molecular polar surface area (mPSA) was calculated for all 2000 molecules from the Spectrum Collection as a crude measure of likely CNS penetrance [71] and a Lipinski Filter was also applied to determine which molecules were most drug-like. This filter applies the “rule of five” [72] which selects compounds with a cLogP <5, molecular mass <500, <5 hydrogen bond donors (OH+NH count), and <10 hydrogen bond acceptors (O plus N atoms).

### Transgenic C57Bl/6 SOD1^G93A^ model of ALS

All mouse experiments were carried out in accord with the Animals (Scientific Procedures) Act 1986 under a UK Home Office project license reviewed by the Sheffield University Ethical Review Committee Project Applications and Amendments Sub-Committee of the Sheffield University Ethical Review Committee and by the Animal Procedures Committee (London, UK). Pharmacology experiments in SOD1^G93A^ transgenic mice were conducted according to the published guidelines for studies in this model [31]. Mice were originally obtained from the Jackson Laboratory, B6SJL-Tg (SOD1-G93A)1Gur/J (stock number 002726), and were subsequently backcrossed onto the C57Bl/6 background (Harlan UK, C57Bl/6 J OlaHsd) for >20 generations. Power analysis indicated that 13–15 mice per group would detect a difference in survival ratio of 10% which is widely accepted as biologically relevant and could detect a difference in time for rotarod performance to decline of 1 week. The litters were split to enable an even distribution of sex and parentage between the two comparison groups and the groups randomly assigned to treatment or control. Previous extensive experiments have indicated that this method of generating control groups provides robust data [36]. Our model, on a defined inbred genetic background, shows no effect of sex or litter of origin on survival [36]. Female SOD1^G93A^ C57BL/6J mice were injected with 10 ml/kg of vehicle (0.1% DMSO in water), or *S*[+]-apomorphine at 0.5 mg/ml subcutaneously to give a final dose of 5 mg/kg, daily from 21 days of age through to end-stage (complete loss of righting reflex for more than 10 s). Behavioural assessment was carried out blinded to treatment group. Rotarod training was performed over 3 days with 2 trials per day. Subsequently, the rotarod test was performed once per week in the afternoon. On each day, mice were tested twice with a rest period in between tests. The best score was taken for analysis. The rotarod (Ugo Basile 7650) was set to accelerate from 4 to 40 rpm in 300 s. Latency to fall (s) was recorded in seconds for each mouse. The test was performed until mice reached a time of <5 s. The catwalk gait analysis system (Noldus Instruments, version 7.1) was used to capture gait parameters in groups of 5 G93A—transgenic mice and 7 nontransgenic mice. Mice were tested every 2 weeks from 6 weeks of age to 16 weeks of age. They were placed on the catwalk apparatus in complete darkness and the recording of gait patterns was performed in a separate room. Multiple runs were recorded for each mouse and three selected for analysis. Processing of gait data was performed using the dedicated software, with the assignment of limbs performed manually and subsequent automated calculation of gait parameters.

### Neuromuscular junction staining

Mice were euthanized by overdose of pentobarbitone and perfused with PBS; both gastrocnemius muscles were dissected out and postfixed in 4% paraformaldehyde for 20 min, followed by cryopreservation through 3 successive 5-min incubations in 5, 10, and 15% sucrose. Specimens were incubated overnight in 20% sucrose and then embedded in OCT embedding matrix. Thirty five-micrometer-thick longitudinal sections were collected. Tissue sections were dried, permeabilised in blocking solution (0.5% Triton X-100, 5% BSA in PBS) at 37 °C for 1.5 h, and then incubated with primary antibodies, antineurofilament M (145kD) 1:1000, from Millipore (AB1987), and synaptophysin 1:1000, from Synpatic Systems (101 002), overnight at RT in 1% BSA and 0.25% Tx-100 in PBS. Anti-rabbit Dylight 488 conjugated secondary antibody (Stratech, 71–485–152) was applied on the sections together with α-bungarotoxin-tetramethylrhodamine conjugate both at a 1:1000 dilution in PBS; 1% BSA and incubated for 2 h at room temperature before washing and mounting. Images were captured on an Incell 2000 and innervated (yellow) or denervated (red) end-plates were counted with a minimum of 1000 NMJs counted per animal.

### Human patient fibroblast assays

#### Oxidative stress assay

Fibroblasts were obtained from human controls (*n*=3) and ALS patients with I113T SOD1 mutations (*n*=3) and sporadic ALS patients (*n*=2). Cells were plated at 2500 cells/well in a 96-well plate in 200 µl minimal media (PAA E15–825) supplemented with 10% FCS gold (PAA A15–151), 2 mM glutamine (Lonza BE17–605E), 50 µg/ml uridine (Sigma U3003), 100X vitamins (Lonza 13–607C 5 ml in 500 ml), 100X amino acids(Lonza BE13–114E 5 ml in 500 ml), sodium pyruvate (Lonza BE13–115E 5 ml in 500 ml), and penicillin/streptomycin (Lonza Be16–603E 5 ml in 500 ml 5000 U/ml). The cells were incubated at 37 °C/5% CO_2_ overnight. The following day the media were removed and the cells were washed with 200 µl 1XP BS. Phenol red-free DMEM/F12 media (Sigma D6434) was added in the presence of 10% serum/2 mM Gln (++), 10% serum/0 mM Gln (+−), or 0% serum/0 mM Gln (−−). Cells were incubated at 37 °C/5% CO_2_ for 5 h. A 50-µl volume containing 10 µM carboxy-H_2_DCFDA and 0.34 µM EthD1 in 1X PBS was added for 1 h.

The plate was read at Ex_485 nm_/Em_530 nm_ (carboxy-H_2_DCFDA fluorescence) and Ex_530 nm_/Em_645 nm_ (EthD1, toxicity). To measure cell number the plate was freeze-thawed and 50 µl of 1 µM EthD1 in PBS was added and the fluorescence at Ex_530 nm_/Em_645 nm_ measured. Carboxy-H_2_DCFDA fluorescence was normalised to live cell number (total EthD1 signal after freeze/thaw – EthD1 signal after assay) to compare between the different cell lines. Data were analysed by one-way ANOVA with Bonferroni post tests using GraphPad Prism

#### S[+]-Apomorphine assay

Cells were plated as above and the following day the media were removed and replaced with media containing 3.7 or 0.41 µM *S*[+]-apomorphine (Sigma, DO43) or 0.02% DMSO as a control. The cells were incubated for 24 h at 37 °C/5% CO_2_, and the cells were washed with 200 µl 1X PBS. DMEM/F12 (50 µl) media were added in the presence of 10% serum/0 mM Gln (+−) and the cells were incubated at 37 °C and 5% CO_2_ for 5 h. A 50-µl volume of 10 µM carboxy-H_2_DCFDA in 1X PBS was then added for 1 h and the plate read at Ex_485 nm_/Em_530 nm_. To measure the cell number the plate was freeze-thawed, 5 0ul of 2 µM EthD in PBS was added to each well, and the fluorescence read at Ex_530 nm_/Em_645 nm_.

At the two apomorphine concentrations tested, there was no effect on cell number compared to the DMSO control; therefore data were analysed by removing blank values from the test carboxy-H_2_DCFDA fluorescence values. The data were tested for normality and analysed by one-way ANOVA using GraphPad Prism.

### Pharmacokinetic analysis

Pharmacokinetic studies were performed by Pharmidex (London, UK). All plasma, CSF, and brain samples were analysed by LC/MS-MS using an Agilent 6410 triple quad LC/MS system to quantify the amount of drug present using a spiked standard curve in the corresponding matrix to the samples. All concentrations were calculated with reference to the relevant standard curve. An extraction buffer (EB) solution of acetonitrile with 0.1% formic acid (Fisher Scientific, HPLC grade) and 500 ng/ml of propranolol as the internal standard (IS) was used for in all extraction protocols. A 1-µl volume of samples was run on a Phenomenex Synergi Hydro-RP 30×2.1 mm column on an Agilent G1312B HPLC with mobile phases of methanol + 0.1% formic acid (A) and water + 0.1% formic acid (B), run in a 0.5 ml gradient from 10% A to 95% A at a flow rate of 0.5 ml/min followed by mass spectrometry.

### Pharmacokinetic modeling

A physiologically based pharmacokinetic (PBPK) model for apomorphine was developed in the Simcyp mouse simulator (v11.1) (Simcyp Limited, Sheffield, UK). The following physicochemical data were used: LogP 3.5 (Supplementary Table 2), MW 267.3, compound type monoprotic base (p*K*_a_ 8.92). A plasma protein binding fraction in mouse of 0.222 (unbound) and a blood:plasma ratio of 1 as measured in humans was assumed [73]. The observed iv clearance of apomorphine in the mouse (109 ml/min/kg, supplementary Table 4) was used to describe the elimination of the compound. Using a fixed value for clearance makes the assumption that clearance is linear across the dose ranges studied. Distribution was predicted using the method of Poulin and Theil as corrected by Berezhkovskiy [74]. The measured brain:plasma (*K*_p_) value (2.1) was used in the model and the *K*_p_ values for other tissues were manually adjusted to give a reasonable fit of the observed data. To model exposure after oral dosing a first-order absorption model was used (*F*_a_=1; *K*_a_=1.5) After showing that the model could capture the observed plasma and brain levels after iv and oral dosing, simulations were performed to predict plasma and brain concentrations following a 2.5 and 5 mg/kg subcutaneous dose. To do this an extravascular dose was administered such that the AUC was 2.5 or 5 times higher than that at the 1 mg/kg intravenous dose. This assumes that the fraction absorbed following subcutaneous dosing was 1 as has been observed in studies in humans [75]. Simulations were performed with a range of *K*_a_ values to give similar *T*_max_ values to those seen following subcutaneous dosing in humans (8–16 min) [76].

### Pharmacodynamic analysis

Male C57BL/6J mice at 6–8 weeks of age were injected with 10 ml/kg of vehicle (0.1% DMSO in water), or *S*[+]-apomorphine at 0.25 or 0.5 mg/ml subcutaneously to give final doses of 2.5 and 5 mg/kg. Tissue was collected from groups of three mice for each dose at 6, 24, 48, and 84 h into RNA later. RNA was isolated using Qiagen RNeasy mini kit and Q-RTPCR performed on HO-1 and NQO-1 genes as described previously.

### Statistical analysis

For the Spectrum library screen, each 384-well plate contained 32 wells incubated with vehicle only (0.1% DMSO in media). The average fluorescence reading and standard deviation of these wells was calculated on a plate by plate basis. Hits were classified as having a GFP fluorescence value greater than the vehicle average plus three standard deviations. Toxicity was defined in the same way (i.e., EthD1 fluorescence value greater than the vehicle average plus three standard deviations).

## Competing Interests

R.J.M., A.H., S.C.B., and P.J.S. are named inventors on a patent relating to use of *S*[+]-apomorphine in ALS.

## Figures and Tables

**Table 1:** Profile of 17 best hit compounds with antioxidant or neutral effects in NSC34 cells following serum withdrawal.

Name	NSC34 oxidative stress assay			1321N1 oxidative stress assay			C6 oxidative stress assay			Calculated properties	
	IC_50_ (μM)	Max reduction (%)	Toxic dose (μM)	IC_50_ (μM)	Max reduction (%)	Toxic dose (μM)	IC_50_ (μM)	Max reduction (%)	Toxic dose (μM)	ALogP	mPSA
2,6-Dihydroxy-4-methoxytoluene	~3.26	28	None	NA	40	None	5.29	16	10	1.815	49.68
Apomorphine hydrochloride	0.174	25	10	1.7	79	None	1.69	62	None	3.498	43.7
4-Methoxychalcone	NA	24	None	3.6	30	None	3.55	82	None	3.685	26.3
Securinine	NA	20	None	18.3	55	None	0.645	67	None	1.448	29.53
Levulinic acid	NA	15	None	3.31	58	None	7.69	62	None	1.652	54.37
3-α-Hydroxygedinin	NA	14	10	NA	25	None	3.22	42	None	3.83	85.97
Hydroquinone	NA	0	None	3.38	80	None	7.07	67	None	1.346	40.46
Andrographolide	NA	0	10	0.501	35	10	0.192	69	10	2.056	86.99
Deacetylgedunin	NA	0	None	2.37	28	10	5.36	70	None	2.97	89.26
3β Hydroxydeoxydesacetoxy-7-oxogedunin	NA	0	3	3.96	62	None	3.07	67	None	2.683	89.26
Isogedunin	NA	0	1	NA	10	None	NA			3.349	95.34
Deoxyandirobin lactone	NA	0	None	1.64	65	None	2.87	38	None	3.399	85.97
Parthenolide	NA	0	1	28.4	47	None	3.11	81	None	2.923	38.82
Epoxy(4,5α)-4,5-dihydrosantonin	NA	0	3	0.662	63	None	2.13	62	None	1.598	55.89
3 Acetoxypregn-16-en-12,20-dione	NA	0	None	12.4?	75	None	5.34	50	None	3.213	60.44
Thymoquinone	NA	0	None	11	25	None	0.014	32	None	2.288	34.14
Dalbergione	NA	0	None	64	38	None	46	40	None	2.94	34.14

The 44 hits from the library screen were assayed in a motor neuronal cell line (NSC34) for ability to protect the cells from a 6-h oxidative stress insult (serum withdrawal) after a 24-h preconditioning with compound at various concentrations. Similar assays were utilised in two distinct astrocyte cell lines (1321N1 and C6). In addition, calculated physical/chemical properties (molecular polar surface area and ALogP) were determined using Pipeline Pilot. Maximum % inhibition of oxidative stress measured by DCF fluorescence and IC_50_ for the inhibition are shown. In addition the minimum dose at which toxicity was observed is shown. NA, not applicable (insufficient data, no concentration response or no inhibition). Compounds are sorted by protective capacity in the NSC34 cell line. ARE inducers were far more effective at protecting the astrocyte cell lines (1321N1 and C6) from oxidative stress compared to NSC34 cells. In order to select compounds for further assessment the following criteria were applied: protective or neutral effect in the NSC34 cell line, AlogP>1, <4, mPSA<100, lack of known toxicity in vivo.

## Footnotes

Appendix A Supplementary data associated with this article can be found in the online version at doi:10.1016/j.freeradbiomed.2013.04.018.
