# Supplementary material for: S[+] Apomorphine is a CNS penetrating activator of the Nrf2-ARE pathway with activity in mouse and patient fibroblast models of amyotrophic lateral sclerosis
Source: Free Radic Biol Med. 2013 Aug;61:438–52. doi: 10.1016/j.freeradbiomed.2013.04.018 (PMC3684770; doi:10.1016/j.freeradbiomed.2013.04.018)
Supplement: Supplementary file 1 — Supplementary Material [file mmc1.doc]

**Supplementary Results**

**Reporter Assay Validation**

The 4xARE-TK-GFP and TK-GFP reporter cell lines were tested for their response to the known ARE inducers tert-butyl hydroquinone (tBHQ) and the flavonoid epigallocatechin gallate (EGCG) at a range of concentrations. The compounds were applied in triplicate to confluent cells in 96 well plates in serum free medium for 24 hours and the induction of GFP measured in a fluorescence plate reader. Both compounds induced GFP expression in a narrow window, with C_max_ (concentration inducing a maximal response) of 100μM for EGCG and 10μM for tBHQ (**Supplementary Figure 1a**). At higher concentrations, both compounds showed signs of toxicity by direct observation (cell loss) or increased ethidium homodimer fluorescence (data not shown) resulting in a reduction in the GFP reporter signal. No increase in fluorescence was seen in the control TK-GFP cell line (data not shown).

**Drug library screening in CHO reporter cell lines**

In order to screen the Spectrum collection of 2000 molecules the reporter assay was optimised in 386 well-plates. To assess the suitability of the assay for library screening, a Z’ score calculation was performed by treating alternate wells with vehicle (0.1% DMSO) and 10 μM Ebselen as a positive control (see calculation in Methods). We have previously shown Ebselen gives a robust response in this assay *(28)*. The calculated Z’ score was 0.51 (**Supplementary** **Figure 1B**) which is acceptable for library screening. In addition, signal to noise (S/N) and signal to background (S/B) ratios were acceptable at 12.8 and 2.9 respectively. The library was subsequently screened at a single point concentration of 10μM for every compound. Drug library dilutions and plating were performed with a Q-BOT liquid handling system and both the 4xARE-TK-GFP reporter cell line and TK-GFP control cell line were tested for their response to the compounds. Hits were identified as having data points more than three standard deviations above the background level, which was the average value of 24 wells treated with vehicle (0.1% DMSO) only. Hit compounds were checked to see if they generated a response in the control cell line, for example by non-specific activation of transcription or autofluorescence. Any compounds which showed such activity were classified as false positives. The library screen was repeated once in the 4xARE –TK-CHO cell line and compounds classed as hits in both screens were taken forward for further assessment. A total of 44 compounds were identified on

this basis.

**Supplementary Table 1** gives the results for all 44 compounds in the primary screening assay.

**Supplementary Table 2** gives the anti-oxidant activities in a motor neuron cell line (NSC34) and two different astrocyte cell lines (1321N1 and C6) as well as key calculated properties for all 44 hit compounds.

**Supplementary Table 3** gives the full pharmacokinetic profile for S[+] apomorphine following intravenous and oral dosing in C57BL/6 mice.

**Supplementary Figure 1.**

Primary assay validation. (**A)** Concentration response curves for tBHQ (closed squares) and EGCG (closed circles) in the CHO-4xARE-TK cell line. Both molecules have a narrow window of ARE activation peaking at 10μM and 100μM respectively. (**B**) results of Z’ score determination. The average +/- SD for vehicle and 10μM Ebselen wells (192 wells each) from a single 384 well plate are shown. The Z’ score for this assay was 0.51 with signal to noise (S/N) and signal to background (S/B) ratios of 12.8 and 2.9 respectively.


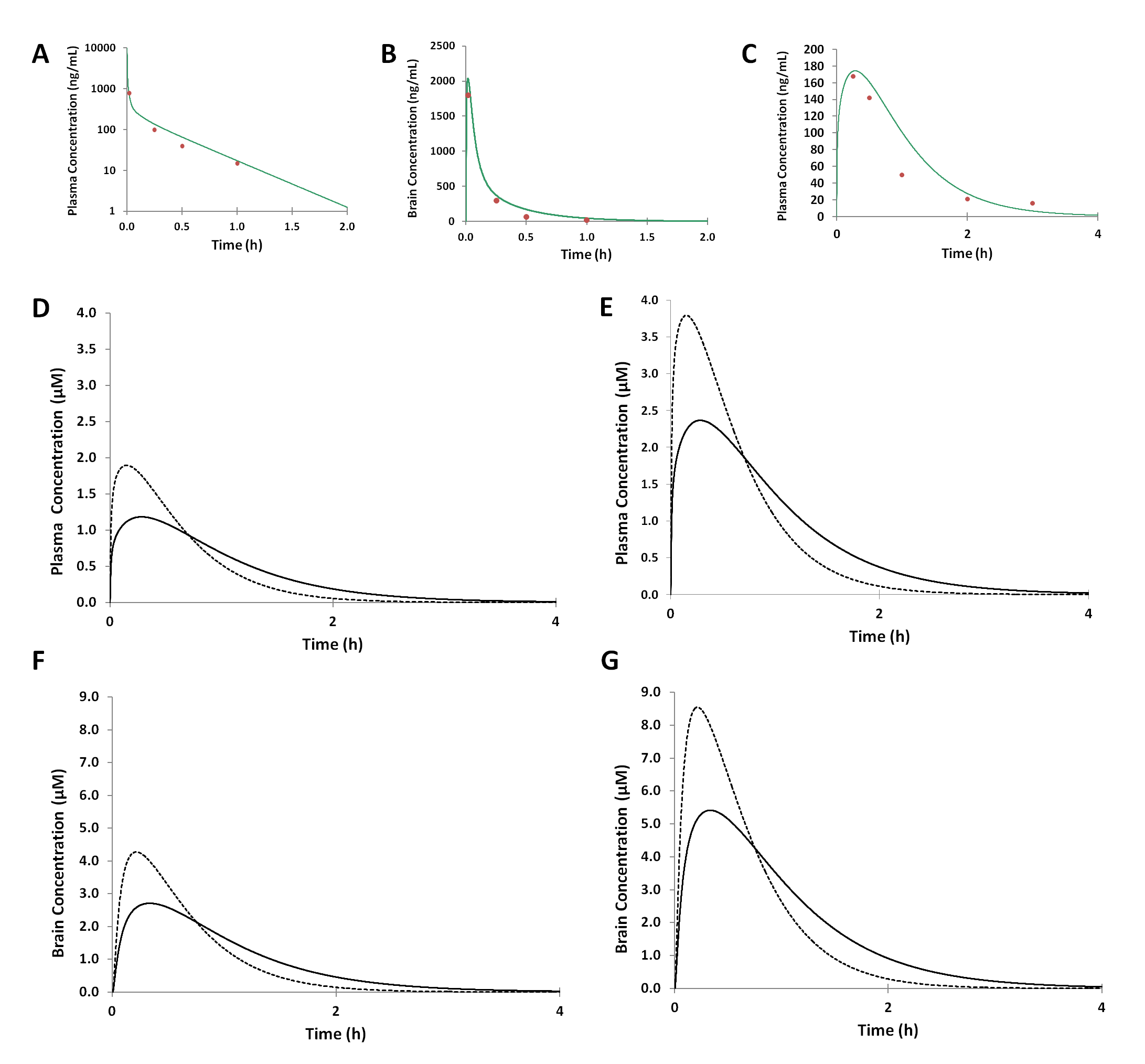


**Supplementary Figure 2.**

Predicted (line) and observed (symbols) concentrations in plasma (A) and brain (B) following

intravenous dosing (1 mg/kg) and in plasma (C) following oral dosing (10 mg/kg).

Simulations of (S)-apomorphine concentrations in plasma (D and E) or brain (F and G) following sub-cutaneous dosing of 2.5mg/kg (D, F) or 5mg/kg (E, G). Each simulation was conducted using a Ka of either 1.5 (solid line) or 3 (dashed line). Tmax was 0.28h and 0.32h in plasma and brain, respectively when Ka was 1.5. Tmax was 0.14h and 0.2h in plasma and brain, respectively, when Ka was 3.

| **Compound name** | **Bioactivity and/or source** | **^1^Fold GFP induction** | **^2^EC_50_ (μM)** | **^3^Toxic dose (μM)** |
| --- | --- | --- | --- | --- |
| Andrographolide (diterpenoid) | Natural product used in Indian and Chinese Medicine | 3.61 | 0.74 | 100 |
| Deoxygedunin (triterpenoid) | Similar to known anti-malarial | 1.82 | 1.15 | 30 |
| Propachlor | Pesticide, high dose neurotoxic | 3.25 | 1.49 | 100 |
| Securinine | GABAA receptor antagonist, alkaloid, CNS stimulant | 3.00 | 2.12 | 30 |
| Dalbergione | Natural product | 2.09 | 2.47 | 30 |
| Isoliquiritigenin | Licorice flavanoid, aldose reductase inhibitor | 2.58 | 2.52 | 30 |
| 4 acetoxyphenol | Natural product, antioxidant | 2.78 | 2.91 | 100 |
| 3 acetoxypregn-16-en-12,20-dione | Natural product derivative | 3.14 | 2.98 | 30 |
| 2 methyl gramine | Natural product derivative | 2.27 | 3.00 | 100 |
| Hydroquinone | Skin whitener, antioxidant | 2.95 | 3.07 | 100 |
| Alachlor | Herbicide, high dose neurotoxic | 3.50 | 3.29 | 100 |
| Kinetin riboside | Purine derivative | 1.97 | 3.39 | 30 |
| Deacetylgedunin | Natural Product | 3.05 | 3.50 | 30 |
| Sappanone A trimethyl ether | Natural product derivative | 1.99 | 3.50 | 100 |
| Swietenolide 3 acetate | Swieteni mahagoni seeds, antifungal | 2.82 | 3.50 | 100 |
| Ebselen | Antioxidant | 2.81 | 4.00 | 100 |
| Citrinin | Natural product, antibacterial | 3.26 | 4.10 | 30 |
| Mechloroethamine | Antineoplastic, alkylating agent | 2.25 | 5.50 | 30 |
| Isoxsuprine hydrochloride | Vasodilator | 1.52 | 8.70 | 100 |
| 3β hydroxydeoxydesacetoxy-7-oxogedunin | Natural Product | 2.55 | 8.71 | 30 |
| Chlordane | Pesticide | 1.56 | 9.18 | 100 |
| 3 methylorsellinic acid | Benzoic derivative, pospholipase A2 inhibitors | 2.87 | 9.59 | 100 |
| Pipobroman | Antineoplastic, alkylating agent | 3.21 | 10.0 | 100 |
| Deoxyandirobin lactone | Semisynthetic | 3.03 | 11.1 | 100 |
| Levulinc acid | Natural product derivative | 2.94 | 12.0 | None |
| Ethacrynic acid | Diuretic | 2.72 | 14.0 | 100 |
| Penicillic acid | Fungal toxin, antibacterial | 2.64 | 14.9 | 100 |
| 4 methoxychalcone | Natural product | 2.13 | 15.0 | 100 |
| 3 methoxycatechol | Machaerium kuhlmanni | 3.28 | 15.4 | 100 |
| Lobaric acid | Natural product | 2.53 | 17.0 | 100 |
| Epoxy(4,5α)-4,5-dihydrosantonin | Anti parasitic, toxicity affects vision | 2.91 | 18.2 | 100 |
| endecaphyllin X | Nitrogenous glucoside miserotoxin | 2.15 | 18.8 | None |
| Benzyl isothiocyanate | Natural product, antineoplastic, antibacterial, antifungal | 1.81 | 24.0 | None |
| Apomorphine hydrochloride | Dopamine agonist | 1.90 | 25.9 | 100 |
| 2,6-dihydroxy-4-methoxytoluene |  | 3.05 | 45.0 | 100 |
| Parthenolide | NSAID,HCV inhibitors | 2.77 | >100 | 100 |
| Isogedunin | Natural Product | 3.11 | >100 | None |
| Methyl-7-deshydroxypyrogallin-4-carboxylate | Synthetic analog antioxidant | 2.83 | >100 | 100 |
| 4-Methoxydalbergione | Natural product | 2.11 | NA | 100 |
| Antiarol | Natural product | 1.64 | NA | 100 |
| Thymoquinone | Natural product | 2.58 | NA | 30 |
| Euphol acetate | Natural product | 2.20 | NA | 100 |
| Retusoquinone | Natural product | 2.04 | NA | 100 |
| 3-alpha-hydroxygedinin | Natural product | 3.01 | NA | 100 |

**Supplementary Table 1.**

Hit ARE-inducing compounds assessed in CHO cells from the Spectrum library screen. Compounds which showed activity in both library screens and lacked toxicity and activity in the control cell lines were assayed twice in a 7-point concentration response curve to determine average EC_50_  and fold-GFP induction. The lowest dose at which toxicity occurred, assessed by a reduction in reporter signal intensity, is also included. Compounds are ranked by EC_50_. ^1^Fold GFP induction is the average fold GFP induction over baseline at the maximal response. ^2^EC_50_ (μM) is the concentration required to give a 50% response. ^3^Toxic dose is the lowest dose at which a reduction in GFP fluorescence was observed. NA, not applicable-EC_50_ could not be estimated due to inability to fit a concentration response curve.

**Supplementary Table 2**

**Supplementary Table 2.** Anti-oxidant activities and physical/chemical properties of all hit compounds. The 44 hits from the library screen were assayed in a motor neuronal cell line (NSC34) for ability to protect the cells from a 6 hour oxidative stress insult (serum withdrawal) after a 24h pre-conditioning with compound at various concentrations. Similar assays were utilised in two distinct astrocyte cell lines (1321N1 and C6). In addition, calculated physical/chemical properties (molecular polar surface area and ALogP) were determined using Pipeline Pilot. Maximum % inhibition of oxidative stress measure by Carboxy-H_2_DCFDA fluorescence and IC_50_ for the inhibition are shown. In addition the minimum dose at which toxicity was observed is shown. NA, not applicable (insufficient data, no concentration response or no inhibition) Compounds are sorted by protective capacity in the NSC34 cell line. ARE inducers were far more effective at protecting the astrocyte cell lines (1321N1 and C6) from oxidative stress compared to NSC34 cells. In order to select compounds for further assessment the following criteria were applied; protective or neutral effect in the NSC34 cell line, AlogP>1, <4, mPSA<100, lack of known toxicity in vivo. Compounds which passed these criteria are shown in bold. In addition, the Lipinski filter was used to identify non-drug like molecules and four were excluded (swietenolide-3-acetate, endecaphylin X, lobaric acid and euphol acetate). *4-acetoxyphenol also showed pro-oxidant activity in this assay.

|  | NSC34 oxidative stress assay | | | 1321N1 oxidative stress assay | | | C6 oxidative stress assay | | | Calculated properties | |
| --- | --- | --- | --- | --- | --- | --- | --- | --- | --- | --- | --- |
| Name | IC_50_ (μM) | Max reduction (%) | Toxic dose (μM) | IC_50_ (μM) | Max reduction (%) | Toxic dose (μM) | IC_50_ (μM) | Max reduction (%) | Toxic dose (μM) | ALogP | mPSA |
| Pipobroman | NA | 30 | none | 3.16 | 19 | 10 | 11 | 71 | none | 0.647 | 40.62 |
| **2,6-Dihydroxy-4-methoxytoluene** | **~3.26** | **28** | **none** | **NA** | **40** | **none** | **5.29** | **16** | **10** | **1.815** | **49.68** |
| **Apomorphine hydrochloride** | **0.174** | **25** | **10** | **1.7** | **79** | **none** | **1.69** | **62** | **none** | **3.498** | **43.7** |
| **4-Methoxychalcone** | **NA** | **24** | **none** | **3.6** | **30** | **none** | **3.55** | **82** | **none** | **3.685** | **26.3** |
| **Securinine** | **NA** | **20** | **none** | **18.3** | **55** | **none** | **0.645** | **67** | **none** | **1.448** | **29.53** |
| **Levulinic acid** | **NA** | **15** | **none** | **3.31** | **58** | **none** | **7.69** | **62** | **none** | **1.652** | **54.37** |
| **3-Alpha-hydroxygedinin** | **NA** | **14** | **10** | **NA** | **25** | **none** | **3.22** | **42** | **none** | **3.83** | **85.97** |
| Penicillic acid | NA | 9 | none | NA | 45 | none | 4.62 | 68 | none | 0.778 | 55.76 |
| Deoxygedunin | NA | 7 | 10 | 1.41 | 25 | 10 | 2.19 | 61 | none | 4.217 | 82.81 |
| **Hydroquinone** | **NA** | **0** | **none** | **3.38** | **80** | **none** | **7.07** | **67** | **none** | **1.346** | **40.46** |
| **Andrographolide** | **NA** | **0** | **10** | **0.501** | **35** | **10** | **0.192** | **69** | **10** | **2.056** | **86.99** |
| **Deacetylgedunin** | **NA** | **0** | **none** | **2.37** | **28** | **10** | **5.36** | **70** | **none** | **2.97** | **89.26** |
| **3β Hydroxydeoxydesacetoxy-7-oxogedunin** | **NA** | **0** | **3** | **3.96** | **62** | **none** | **3.07** | **67** | **none** | **2.683** | **89.26** |
| **Isogendunin** | **NA** | **0** | **1** | **NA** | **10** | **none** | **NA** |  |  | **3.349** | **95.34** |
| **Deoxyandirobin lactone** | **NA** | **0** | **none** | **1.64** | **65** | **none** | **2.87** | **38** | **none** | **3.399** | **85.97** |
| Endecaphyllin X | NA | 0 | 10 | NA | 56 | none | 3.15 | 40 | none | -9.6E-02 | 317.93 |
| **Parthenolide** | **NA** | **0** | **1** | **28.4** | **47** | **none** | **3.11** | **81** | **none** | **2.923** | **38.82** |
| Alachlor | NA | 0 | 10 | 1.97 | 83 | none | 6.22 | 62 | none | 3.425 | 29.54 |
| Propachlor | NA | 0 | 10 | 0.829 | 50 | 10 | 0.645 | 74 | 10 | 2.415 | 20.31 |
| Chlordane | NA | 0 | 3 | NA | 68 | none | 3.57 | 20 | none | 4.97 | 0 |
| **Epoxy(4,5α)-4,5-dihydrosantonin** | **NA** | **0** | **3** | **0.662** | **63** | **none** | **2.13** | **62** | **none** | **1.598** | **55.89** |
| Kinetin riboside | NA | 0 | 3 | 0.246 | 25 | 3 | 3.84 | 40 | 10 | -1.033 | 138.68 |
| **3 Acetoxypregn-16-en-12,20-dione** | **NA** | **0** | **none** | **12.4?** | **75** | **none** | **5.34** | **50** | **none** | **3.213** | **60.44** |
| **Thymoquinone** | **NA** | **0** | **none** | **11** | **25** | **none** | **0.014** | **32** | **none** | **2.288** | **34.14** |
| Citrinin | NA | 0 | none | 0.338 | 28 | none | 20 | 23 | none | 0.928 | 83.83 |
| **Dalbergione** | **NA** | **0** | **none** | **64** | **38** | **none** | **46** | **40** | **none** | **2.94** | **34.14** |
| Swietenolide 3 acetate | NA | 0 | 0.3 | NA | 60 | none | 4.22 | 50 | none | 2.633 | 129.34 |
| Isoliquiritigenin | NA | -6 | 1 | 1.78 | 50 | none | 28? | 46 | none | 2.976 | 77.76 |
| Mechloroethamine | NA | -10 | 10 | 0.302 | 37 | 10 | 3.42 | 67 | none | 1.65 | 3.24 |
| 3-Methylorsellinic acid | NA | -12 | 0.3 | 3.26 | 53 | none | 3.1 | 56 | none | 1.948 | 77.76 |
| 3-Methoxycatechol | NA | -13 | 0.3 | NA | 39 | none | 1.2 | 46 | 10 | 1.329 | 49.68 |
| Isoxsuprine hydrochloride | NA | -16 | none | NA | ? | none | 23 | 40 | none | 3.384 | 61.71 |
| Methyl-7-deshydroxypyrogallin-4-carboxylate | NA | -23 | 0.3 | NA | 45 | none | 2.95 | 70 | none | 1.133 | 104.06 |
| Retusoquinone | NA | -25 | none |  | 0 | none | 2.06 | 15 | none | 2.684 | 17.07 |
| Lobaric acid | NA | -26 | none |  | 0 | none | 3.14 | 9 | none | 5.993 | 119.36 |
| Benzyl isothiocyanate | NA | -28 | none | NA | 8 | none | 3.47 | 27 | none | 2.596 | 44.45 |
| Ethacrynic acid | NA | -30 | 10 | 3.01 | 25 | none | 3.01 | 31 | none | 3.964 | 63.6 |
| 2-Methyl gramine | NA | -30 | 3 | 11 | 36 | none | 32 | 36 | none | 2.342 | 19.03 |
| Euphol acetate | NA | -43 | none | 18 | 38 | none | NA | 0 | none | 8.498 | 26.3 |
| 4-Methoxydalbergione | NA | -44 | none | NA | 43 | none | 2.5 | 14 | none | 2.627 | 43.37 |
| Antiarol | NA | -50 | 10 | NA | 35 | none | 3.05 | 23 | 10 | 1.538 | 47.92 |
| Sappanone A trimethyl ether | NA | -60 | none | 0.279 | 40 | none | NA | 27 | none | 3.35 | 53.99 |
| 4-Acetoxyphenol | NA | -70 | none | 0.298 | 42* | none | NA | 0 | none | 1.355 | 46.53 |
| Ebselen | NA | -80 | none | 0.193 | 20 | none | NA | 30 | none | 3.227 | 20.31 |

**Supplementary Table 3**

**Supplementary Table 3.**

Pharmacokinetic parameters calculated following dosing of mice with 10mg/kg (oral, po) or 1mg/kg (intravenous, iv) S[+] apomorphine and analysis of plasma (po, iv) brain tissue, (Iiv) and cerebrospinal fluid (CSF, iv) by LC-MS-MS in 3 (po) or 4 (iv) mice per timepoint at 6 timepoints post-dosing. S[+] apomorphine shows high brain uptake and a short half-life.
